# Influenza Management During the COVID-19 Pandemic: A Review of Recent Innovations in Antiviral Therapy and Relevance to Primary Care Practice

**DOI:** 10.1016/j.mayocpiqo.2021.07.005

**Published:** 2021-08-14

**Authors:** Warren A. Jones, Rita de Cassia Castro, Henry L. Masters, Ruth Carrico

**Affiliations:** a Department of Family Medicine, University of Mississippi Medical Center, Jackson; b Genentech, Inc., San Francisco, CA; c Division of Infectious Diseases, University of Louisville School of Medicine, Louisville, KY

**Keywords:** CDC, Centers for Disease Control and Prevention, COVID-19, coronavirus disease 2019, FDA, Food and Drug Administration, RIDT, rapid influenza diagnostic test, SARS-CoV-2, severe acute respiratory syndrome coronavirus 2

## Abstract

Seasonal influenza requires appropriate management to protect public health and resources. Decreasing the burden of influenza will depend primarily on increasing vaccination rates as well as prompt initiation of antiviral therapy within 48 hours of symptom onset, especially in the context of the current coronavirus disease 2019 pandemic. A careful approach is required to prevent health services from being overwhelmed by a surge in demand that could exceed capacity. This review highlights the societal burden of influenza and discusses the prevention, diagnosis, and treatment of influenza as a complicating addition to the challenges of the coronavirus disease 2019 pandemic. The importance of vaccination for seasonal influenza and the role of antiviral therapy in the treatment and prophylaxis of seasonal influenza, including the most up-to-date recommendations from the Centers for Disease Control and Prevention for influenza management, will also be reviewed.

**Article Highlights**

• Seasonal influenza coupled with coronavirus disease 2019 (COVID-19) represents a dual challenge to the medical community, specifically to frontline health care professionals.
• In addition to vaccination, appropriate influenza disease management and prompt antiviral therapy, ideally within 48 hours of symptom onset, will be imperative in decreasing the burden of influenza, especially in the context of COVID-19.
• The US Centers for Disease Control and Prevention has updated its recommendations on antiviral treatment and recommends antiviral treatment as early as possible (within ≤48 hours) for priority patients: any patient with confirmed or suspected influenza who is hospitalized or has severe, complicated, or progressive illness or is at higher risk for influenza complications.
• This article describes key information on influenza disease management in the context of the COVID-19 pandemic, as well as current antiviral drugs approved for the treatment and postexposure prophylaxis of influenza, including baloxavir marboxil, the first US Food and Drug Administration–approved single-dose selective inhibitor of influenza cap-dependent endonuclease.

With the emergence of the coronavirus disease 2019 (COVID-19) pandemic, the medical community needs to prepare for the management of seasonal influenza and consider the coexistence of COVID-19. The complexities of prevention, diagnosis, management, and capacity across medical services have the potential to be exacerbated by the arrival of influenza season. Primary care clinicians have a critical role in the frontline management of both infections, to protect public health and prevent health services from being overwhelmed.

## Disease Overview

Influenza is an acute respiratory infection with variable degrees of systemic symptoms that can include fever, cough, sore throat, runny or stuffy nose, muscle or body aches, headaches, fatigue, vomiting, and diarrhea.^1^^,^^2^ Although influenza can present with wide-ranging symptomology, an additional 4% to 28% of influenza-infected patients are estimated to be asymptomatic.^3^

Of the 4 types of influenza viruses, types A and B are responsible for causing yearly epidemics of respiratory disease and, occasionally, pandemics.^1^^,^^4^^,^^5^ Influenza is transmitted mainly via respiratory droplets but may also be transmitted by direct contact with an infected person or indirect contact via surfaces.^1^^,^^6^

## Influenza Burden of Disease

Influenza leads to substantial direct and indirect health care burdens, which vary depending on the health status of the infected individual and the treatment setting. Most individuals with uncomplicated influenza have mild, self-limiting illness and recover in less than 2 weeks.^1^^,^^4^ However, for some patients, particularly those in high-risk groups, influenza can lead to more serious complications, including sinusitis, pneumonia, myocarditis, encephalitis, myositis, rhabdomyolysis, respiratory and kidney failure, and sepsis,^2^ which can lead to hospitalization and sometimes death. Influenza can also exacerbate underlying chronic health conditions, with serious consequences. High-risk groups include individuals aged 65 years and older; people with diabetes, asthma, heart disease, stroke, HIV, or cancer; children with neurologic conditions; and women who are pregnant.^4^^,^^7^ Other groups including non-Hispanic Black persons, Hispanic or Latino persons, American Indians and Alaska Natives, children aged less than 5 years, people who live in long-term care facilities, and postpartum women are also considered to be at high risk.^1^^,^^8^

Chronic health problems that can be exacerbated by influenza infection include, but are not limited to, emphysema, chronic bronchitis, asthma, ischemic heart disease, and congestive heart failure.^6^ People with preexisting medical conditions, including conditions that compromise airway clearance, asthma, chronic pulmonary, kidney, or heart disease, immunosuppression, long-term aspirin therapy in patients younger than 19 years of age, metabolic disorders, obesity, sickle cell anemia and other hemoglobinopathies, among others, are at increased risk of influenza-related complications.^1^^,^^8^

Recent Centers for Disease Control and Prevention (CDC) estimates of the annual burden of influenza are 9 to 45 million cases and 12,000 to 61,000 deaths annually since 2010.^9^ Influenza-associated hospitalizations are highest in adults aged 65 years and older, and 90% of influenza-related deaths occur among those aged 65 years and older.^10^^,^^11^

Influenza continues to be associated with substantial economic costs, despite the availability of influenza vaccination. The most up-to-date economic analyses estimate that the average total economic burden of influenza to the US health care system is $3 billion annually, potentially increasing up to $11 billion annually to society when indirect costs, such as lost productivity, are included.^12^ Although total direct costs are disproportionately carried by those older than 65 years of age, primarily due to hospitalization, the impact of indirect costs, resulting from lost income in working-age adults (18 to 49 years) due to influenza-related mortality, should not be discounted. The majority of indirect costs were driven by lost income in working-age adults, which included 20.1 million days of lost productivity for a total of $8 billion in indirect costs.^12^, ^13^, ^14^

## Influenza and COVID-19

Influenza and severe acute respiratory syndrome coronavirus 2 (SARS-CoV-2) are contagious pathogens that cause respiratory illness; these 2 viruses will coexist during influenza season and have several overlapping clinical features but require different approaches to management.^15^ Symptoms common to both COVID-19, caused by SARS-CoV-2, and influenza include fever, cough, shortness of breath/difficulty breathing, fatigue, sore throat, runny or stuffy nose, muscle or body aches, headaches, and vomiting and diarrhea.^15^^,^^16^

Patients with influenza do not typically experience anosmia or dysgeusia, which are sometimes symptoms of COVID-19; these symptoms may take longer to develop than influenza symptoms (2 to 14 days compared with 1 to 4 days, respectively).^15^ Moving forward, clinicians will have to consider influenza virus infection, SARS-CoV-2 infection, and coinfection when making clinical decisions. Diagnostic testing can help to distinguish influenza from SARS-CoV-2 infection and subsequently inform clinical management. Accurate diagnosis is of particular importance for patients with suspected influenza who are being admitted to an emergency department or to determine the cause of a respiratory illness outbreak within a closed setting. However, the CDC indicates that clinicians should not wait for the results of influenza and/or SARS-CoV-2 testing to initiate empirical antiviral treatment for influenza in priority groups (ie, hospitalized patients, patients with severe, complicated, or progressive illness, or those at higher risk for influenza complications).^17^ In addition, during periods of cocirculation of influenza and SARS-CoV-2 within the community, clinicians may consider, based on clinical judgement, early initiation of empirical antiviral treatment of non–high-risk outpatients with suspected influenza, which may include those assessed and evaluated via telemedicine.^17^ The risk of exceeding the capacity of health care facilities has been a serious concern during the COVID-19 pandemic and will continue to be an issue in the coming months. Most recent estimates from the CDC for the 2019-2020 influenza season report 18 to 26 million medical visits, 410,000 to 740,000 hospitalizations, and 24,000 to 62,000 deaths due to influenza.^18^ Therefore, early diagnosis, effective prevention, and management of influenza and influenza-related complications will be crucial to decrease hospital bed usage.^19^ Although diagnostic testing of respiratory infections across the United States was higher than normal during the 2019-2020 influenza season because of the COVID-19 pandemic, the coexistence of influenza and COVID-19 has led to difficulties in accurately attaining estimates for influenza vs influenzalike illnesses in the beginning of the COVID-19 pandemic. Following widespread adoption of community mitigation measures to reduce the transmission of COVID-19, the Southern Hemisphere has experienced low influenza activity in 2020.^20^ In the United States, influenza activity in 2021 continues to be unpredictable, given the varying levels of influenza vaccination patterns and adherence to COVID-19 control measures currently being observed across the country, which may vary geographically over time.

## Prevention

Many of the same mitigation strategies are effective for the prevention of both influenza and COVID-19, including promotion of health habits to prevent viral transmission such as social distancing, staying at home when sick, hand hygiene, and sneeze and cough etiquette. Surgical masks and facial coverings, when used correctly, may also reduce the transmission of influenza and coronavirus in respiratory droplet, and in aerosols in the case of coronarvirus.^21^ Health care professionals have an important role to play in educating their patients on social distancing, mask use, hand hygiene, and cough etiquette. The CDC recommends a combination of infection prevention control strategies that includes covering the nose and mouth when coughing or sneezing, handwashing with nonantibacterial soap and water, and using universal source control such as appropriate use of masks or face coverings.^22^ Patients should be educated to wear a mask or face covering over their nose and mouth and perform hand hygiene before and after touching it.^23^^,^^24^ Because the primary route of influenza transmission is via respiratory droplets, mask wearing may be particularly useful during the upcoming influenza season in public places or where close contact with other persons is expected. This step may be of particular benefit for immunocompromised individuals.

Ensuring the uptake of the seasonal influenza vaccine among eligible individuals will be of utmost importance during the period in which influenza and COVID-19 are expected to cocirculate. Given the novelty of the current COVID-19 pandemic coupled with the uncertainty of continued public health mitigation measures, it is very important to plan for seasonal influenza. Influenza is the most frequent cause of death from a vaccine-preventable disease in the United States, with highest infection rates from seasonal influenza observed among children. However, the risks for complications, hospitalizations, and deaths are higher among adults aged 65 years and older, children younger than 5 years of age, pregnant women, and people of any age who have medical conditions that place them at increased risk for complications from influenza.^25^ Therefore, influenza vaccination for individuals 6 months of age and older remains the best method for influenza prevention, and it is particularly important this influenza season.^13^ These recommendations note that vaccination coverage will be especially important for those at increased risk for severe illness and/or complications from influenza and for influenza-related outpatient/inpatient or emergency department visits.

Influenza vaccination has been estimated to prevent between 1.6 and 6.7 million illnesses, 790,000 to 3.1 million outpatient medical visits, 39,000 to 87,000 hospitalizations, and 3000 to 10,000 deaths from respiratory and circulatory complications related to influenza each season.^26^ Influenza vaccination is the first line of defense against influenza and is of vital importance, particularly in the current environment. However, there are limitations to its effectiveness. The seasonal influenza vaccine is designed to protect against the 3 or 4 influenza viruses that research indicates are *most likely* to spread and cause illness during the upcoming influenza season.^27^ Therefore, the effectiveness of the influenza vaccine varies annually.

Coverage and use of the influenza vaccination at the population level is difficult to manage. During the 2019-2020 influenza season, the CDC estimated vaccination coverage (≥1 dose of influenza vaccine) to be 63.8% among children (aged 6 months to 17 years) and 48.4% in adults older than 18 years or older, which is below national objectives.^28^ This coverage varies greatly between states, from 51.9% to 78.3% in children and 41.4% to 56.8% in adults.^28^ Across the states, lower vaccine coverage in children corresponded to lower vaccination rates among adults. Increasing influenza vaccination coverage in children is especially important because they have been identified as the main spreaders of influenza infection.^29^^,^^30^ Influenza vaccines are not 100% effective, and antiviral treatment may be needed for the management of acute influenza infection.

It is important to note that there is no change in the CDC’s recommendation on timing of vaccination this influenza season. Vaccination in July or August was deemed to be too early, particularly for older people, because of the likelihood of reduced protection against infection later in the influenza season. Instead, September and October were deemed to be more optimal for vaccination. However, the CDC recommends that as long as influenza viruses are circulating, vaccination should continue, even in January or later.^31^ For travelers, eg, those visiting areas of year-round endemic influenza, the CDC recommends vaccination at least 2 weeks before travel.^32^

## Diagnosis and Testing

Both influenza and COVID-19 can result in severe illness and complications, particularly in high-risk individuals; therefore, rapid diagnosis is important to facilitate timely initiation of treatment and allow for appropriate isolation.^15^ Given the overlap in symptoms between influenza and COVID-19, diagnostic testing may be necessary to better inform treatment decisions**.**

In the United States, a number of rapid influenza diagnostic tests (RIDTs) are commercially available for the detection of influenza A and B. The acronym *RIDT* is synonymous with a test that detects an influenza antigen. Historically, these tests have had lower sensitivity than molecular-based tests.^33^ However, in 2017, the US Food and Drug Administration (FDA) reclassified RIDTs from class I to class II devices and now requires specific minimum criteria for sensitivity and specificity.^34^ The RIDTs typically require a nasopharyngeal swab or aspirate and include the 3M Rapid Detection Flu A+B Test, QuickVue Influenza Test (Quidel Corporation), TRU FLU (Meridian Bioscience Inc), and XPECT Flu A&B (Remel Products), among others.^35^ Rapid testing at the point of care is important for early diagnosis and treatment; however, most tests are limited for use in health care settings. Therefore, a number of at-home influenza tests are currently in development for consumer use. Theraflu Home Flu Test (GlaxoSmithKline plc), a novel rapid test based on nasopharyngeal swabs, was found to have good sensitivity and specificity (>86%) for both influenza A and B viruses based on a prospective study of 1012 participants with influenzalike illness conducted at 25 US sites.^36^ The flu@home test (University of Washington School of Medicine) is another nasal swab test that detects influenza A and B; the test kit includes components from the Clinical Laboratory Improvement Amendments (CLIA)–waived Quidel QuickVue Influenza rapid diagnostic test.^37^ The benefits of at-home testing are reduction of the potential for infecting others through in-clinic visits and potential for incorporation of the test results into telehealth appointments and timely antiviral prescription.

Rapid molecular influenza virus testing in the primary/outpatient settings can aid health care professionals in establishing a diagnosis without off-site laboratory analysis.^38^ These relatively new rapid molecular assays detect influenza virus nucleic acids in upper respiratory tract specimens with higher sensitivity (90% to 95%) and specificity than their RIDT counterparts.^33^ The rapid molecular test provides laboratory-quality results, with the advantage that results may be available in approximately 15 to 30 minutes.^39^ Although molecular point-of-care diagnostic testing is preferred to aid health care professionals with appropriate patient management, there is still a high volume of RIDTs being utilized. Thus, the CDC currently recommends that antiviral treatments should not be withheld from priority patients with suspected influenza.^34^ This recommendation is due in part to the low sensitivity of RIDTs (point-of-care antigen test) and the need to give antivirals within 48 hours of the onset of clinical symptoms.

**Figure 1:**
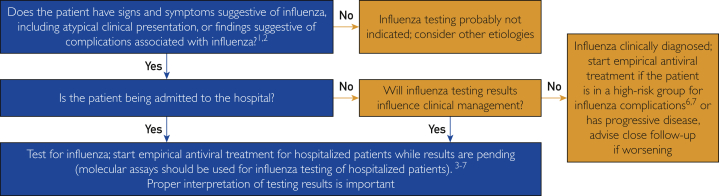
Algorithm for influenza testing during a community outbreak of influenza (Supplemental Appendix). Data from the Centers for Disease Control and Prevention^17^ (freely available at https://www.cdc.gov/flu/professionals/antivirals/summary-clinicians.htm#Treatment). The use of this algorithm adaptation does not imply endorsement of this review article by the Centers for Disease Control and Prevention.

There are several FDA-cleared multiplex nucleic acid amplification tests for influenza viruses.^40^ Furthermore, under Emergency Use Authorization, the FDA has approved several tests that simultaneously detect influenza viruses and SARS-CoV-2 (Table 1).^43^ It should be noted that empirical antiviral treatment should be initiated as soon as possible without waiting for laboratory confirmation of influenza virus infection for priority patients when influenza is known to be circulating in the community (Figure 1; Supplemental Appendix, available online at http://mcpiqojournal.org).^17^ Testing in tandem for these pathogens may be needed in some circumstances for appropriate patient management.

**Table 1:** FDA-Cleared Multiplex Assays for Detection of Influenza and Other Respiratory Viruses (October 2020)

Manufacturer (use)	Product	Platform/instrument	Viruses detected		Complexity
			Influenza	Other respiratory viruses	
BioFire Diagnostics (commercially available)	BioFire Respiratory Panel 2.1 (RP2.1)	FilmArray 2.0 and FilmArray Torch systems	A and B	SARS-CoV-2 Parainfluenza virus 1-4	High Moderate
QIAGEN (commercially available)	QIAstat-Dx Respiratory SARS-CoV-2 Panel	QIAstat Dx Analyzer 1.0 system	A and B	SARS-CoV-2 Parainfluenza virus 1-4	High Moderate
CDC (public health use only, not commercially available)	Influenza SARS-CoV-2 (Flu SC2) Multiplex Assay	Applied Biosystems 7500 Fast Dx Real-Time PCR Instrument	A and B	SARS-CoV-2	High
Roche Diagnostics^41^	cobas SARS-CoV-2 & Influenza A/B	cobas 6800/8800 Systems real-time PCR instrument	A and B	SARS-CoV-2	High
Roche Diagnostics^42^	cobas SARS-CoV-2 & Influenza A/B	cobas Liat System real-time PCR instrument	A and B	SARS-CoV-2	High

CDC, Centers for Disease Control and Prevention; FDA, United States Food and Drug Administration; PCR, polymerase chain reaction; SARS-CoV-2, severe acute respiratory syndrome coronavirus 2.

## Treatment Landscape

**Figure 2:**
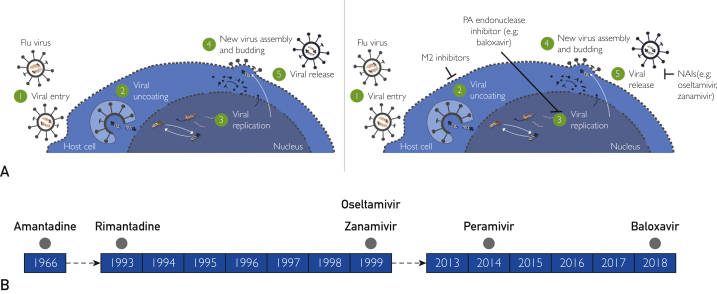
A, Schematic of the influenza life cycle and mechanism of action of influenza antiviral agents. B, Timeline of United States Food and Drug Administration approval of influenza antiviral agents. NAI, neuraminidase inhibitor; PA, polymerase acidic.

A brief overview of the influenza virus life cycle is necessary to understand the mechanism of action of currently available antiviral agents. Influenza belongs to the orthomyxovirus group of RNA viruses. Three major proteins coexist on the surface of the influenza virus: the transmembrane glycoprotein hemagglutinin, which mediates attachment of the virus to the respiratory epithelial cells via specific receptors; neuraminidase, which enzymatically cleaves sialic acid in the host respiratory epithelial cells prior to the release of new virions; and the matrix (M2) protein in influenza A, which forms a protein ion channel facilitating viral entry into the host cell and release of proteins responsible for viral replication.^44^^,^^45^ After viral uncoating within the cell and replication via RNA polymerase, new viral RNA and viral proteins synthesized by the host cell assemble at the cell surface into new virions. After budding, virions are detached from the host cell by viral neuraminidase.^44^^,^^45^ Cross-resistance between different antiviral classes is not expected because they target different viral proteins (Figure 2A).

### M2 Inhibitors

Amantadine and rimantadine are antivirals that target the M2 ion channel of influenza A viruses and therefore are not active against influenza B viruses.^46^ Amantadine was approved by the US FDA in 1966 as prophylaxis against Asian influenza; its derivative rimantadine was approved in 1993 (Figure 2B). These antivirals are no longer recommended because circulating influenza A viruses have developed widespread resistance to these drugs.

### Neuraminidase Inhibitors

The emergence of antiviral resistance is a major challenge in the development of new antiviral drugs.^46^^,^^47^ Neuraminidase inhibitors, which target the enzymatic activity of the viral neuraminidase protein of influenza A and influenza B viruses, include oseltamivir, zanamivir (both approved by the US FDA in 1999), and peramivir (approved in 2014) as the recommended standard of care (Figure 2B).^46^ Neuraminidase inhibitors have been found to be effective for the treatment of influenza in clinical trials and in the real-world setting (Table 2).^59^, ^60^, ^61^ Neuraminidase inhibitors reduce the time to symptom alleviation^54^^,^^56^^,^^57^ and resolution^52^ and reduce the time to cessation of viral shedding.^53^ This class of agents is also generally well tolerated, with mild to moderate gastrointestinal adverse events most frequently reported vs placebo.^52^^,^^54^^,^^57^

**Table 2:** Clinical Efficacy of Antivirals Currently Recommended by the Centers for Disease Control and Prevention for the Treatment of Influenza^a^

Reference, year	Clinical setting	Trial description	No. of patients	Treatment	Primary end point	Primary outcomes
Polymerase acidic endonuclease inhibitor						
Baloxavir						
Hayden et al,^48^ 2018	Uncomplicated influenza in healthy adults and adolescents	Phase 2 R, DB, PC	400	Baloxavir (single dose of 10, 20, or 40 mg) or placebo	Time to alleviation of symptoms	Median time to alleviation of symptoms was shorter in each of the baloxavir dose groups (54.2 h in the 10-mg group, 51.0 h in the 20-mg group, 49.5 h in the 40-mg group) than in the placebo group (77.7 h) (*P*=.009, *P*=.02, and *P*=.005, respectively)
CAPSTONE-1,^48^ 2018	Uncomplicated influenza in healthy adults and adolescents	Phase 3 R, DB, PC, ACC	1436	Baloxavir (single dose of 40 mg for patients weighing <80 kg or 80 mg for those ≥80 kg) or oseltamivir (75 mg bid) or placebo	Time to alleviation of symptoms	Median time to alleviation of symptoms was shorter in the baloxavir group than in the placebo group in the ITT infected population (53.7 h vs 80.2 h; *P*<.001) and ITT population (65.4 h vs 88.6 h; *P*<.001) and similar to the oseltamivir group
Ison et al (CAPSTONE-2),^49^ 2020	Uncomplicated influenza in high-risk adolescent and adult outpatients	Phase 3 R, DB, PC, ACC	2184	Baloxavir (single dose of 40 mg for patients weighing <80 kg or 80 mg for those ≥80 kg) or oseltamivir (75 mg bid) or placebo	Time to improvement of influenza symptoms	Time to improvement of symptoms was shorter in the baloxavir group than in the placebo group (73.2 h vs 102.3 h; *P*<.0001); this difference was significant in patients with influenza A, influenza B, asthma, or chronic lung disease. Time to improvement of symptoms was similar between baloxavir and oseltamivir in patients with influenza A but shorter in patients with influenza B (*P*=.025)
Baker et al (miniSTONE-2),^50^ 2020	Acute influenza in children (1-12 y)	Phase 3 R, DB, ACC	176	Baloxavir (single dose based on weight; 2 mg/kg for those weighing <20 kg and a single dose of 40 mg for those weighing ≥20 kg) or oseltamivir (30 mg for patients weighing ≤15 kg, 45 mg for >15 to ≤23 kg, 60 mg for >23 to ≤40 kg, and 75 mg for >40 kg, bid)	Incidence, severity, and timing of AEs	The overall incidence of AEs was similar between the baloxavir group (46.1%) and the oseltamivir group (53.4%). The incidence of AEs related to study drug was low in both the baloxavir (2.6%) and oseltamivir (8.6%) groups
Ikematsu et al,^51^ 2020	Prophylaxis against influenza in healthy household contacts	R, DB, PC	752	Single, weight-based oral dose of baloxavir or matching placebo. ≥12 y at screening: weight <80 kg, 40 mg; ≥80 kg, 80 mg <12 y at screening: weight <10 kg, 1 mg/kg; 10 to <20 kg, 10 mg; 20 to <40 kg, 20 mg; ≥40 kg, 40 mg	Laboratory-confirmed clinical influenza	Laboratory-confirmed clinical influenza was reduced in the baloxavir group compared with the placebo group (1.9% vs 13.6%, respectively; *P*<.001)
Neuraminidase inhibitors						
Oseltamivir phosphate						
Treanor et al,^52^ 2000	Acute influenza in nonimmunized, previously healthy adults	R, DB, PC	629	Oseltamivir (75 mg or 150 mg bid) or placebo	Time to resolution of illness	Both dose levels of oseltamivir (71.5 h, *P*<.001 [75 mg]; 69.9 h, *P*<.006 [150 mg]) resulted in reductions in the duration of illness vs placebo (103.3 h)
Hayden et al,^53^ 1999	Healthy adult volunteers susceptible to viral influenza challenge	R, DB, PC	117	Prophylaxis study: oseltamivir (100 mg bid or 100 mg qd) or placebo Treatment study: oseltamivir (20, 100, or 200 mg bid or 200 mg qd) or placebo	Frequency of viral shedding and infection	No oseltamivir recipients had recovery of challenge virus from nasal washings (100% efficacy vs 50% in the placebo group; *P*<.001). Median time to cessation of viral shedding was reduced from 107 h in the placebo group to 58 h in the combined oseltamivir group (*P*=.003)
Peramivir						
Kohno et al,^54^ 2010	Previously healthy adults with ILI within the previous 48 h	R, DB, PC	300	Intravenous peramivir (300 mg or 600 mg) or placebo	Time from start of treatment to recovery	Peramivir significantly reduced time to alleviation of symptoms (median 59.1 h, *P*<.0019 [300 mg]; 59.9 h, *P*<.009 [600 mg]) vs placebo (median 81.8 h)
De Jong et al,^55^ 2014	Patients hospitalized with suspected influenza	Phase 3 R, DB, PC	405	Intravenous peramivir (10 mg/kg qd, up to 600 mg/d maximum) or placebo, added to institution’s SOC	Time to clinical resolution	Time to clinical resolution did not differ between peramivir-treated or SOC-only patients (42.5 vs 49.5 h)
Zanamivir						
Monto et al,^56^ 1999	Adults with ILI^b^	Phase 2/3 (pooled data) R, DB, PC	1133	Phase 2 studies: zanamivir (10 mg inhaled qid) vs placebo Phase 3 studies: zanamivir (10 mg inhaled bid) vs placebo^b^	Time to alleviation of major influenza symptoms	Median time to alleviation of influenza symptoms was reduced from 6 d in the placebo group to 5 d in the zanamivir group (*P*<.001)
Hedrick et al,^57^ 2000	Children between the ages of 5 and 12 y with ILI	R, DB, PC	471	Zanamivir (10 mg bid) or placebo	Time to alleviation of clinically significant symptoms of influenza	Zanamivir significantly shortened median time to alleviation of symptoms vs placebo by 24% (4.0 vs 5.25 d; *P*<.001)
LaForce et al,^58^ 2007	Community-dwelling, high-risk adults and adolescents	R, DB, PC	3363	Zanamivir (10 mg qd) or placebo	Proportion of randomized patients in whom symptomatic influenza A or B developed during prophylaxis	4/1678 (0.2%) Zanamivir-treated individuals had development of symptomatic culture/serology–confirmed influenza vs 23/1685 (1.4%) placebo recipients (*P*<.001), with an 83% protective efficacy

a ACC, active comparator controlled; AE, adverse event; bid, twice daily; DB, double-blind; ILI, influenzalike illness; PC, placebo-controlled; qd, once daily; qid, 4 times daily; R, randomized; SOC, standard of care; ITT, intention-to-treat.

b See individual studies for full treatment information.

### Endonuclease Inhibitor

Baloxavir marboxil is the first selective inhibitor of influenza cap-dependent endonuclease, receiving FDA approval in 2018 (Figure 2B) and has been found to have efficacy at least similar to the current standard of care against influenza A and influenza B infections. A single oral dose of baloxavir is effective against neuraminidase-resistant influenza strains.^46^ Baloxavir had superior efficacy to placebo in the phase 3 CAPSTONE trials and comparable efficacy to oseltamivir in the phase 3 CAPSTONE^48^^,^^49^ and miniSTONE-2 trials in otherwise healthy children and adults, including high-risk populations.^50^ The FLAGSTONE trial in severely ill, hospitalized patients with influenza found that baloxavir plus standard-of-care neuraminidase inhibitor was not associated with a significant improvement of clinical outcomes vs neuraminidase inhibitor alone.^62^ Additional studies of baloxavir are under way as part of a phase 3 development program, including an ongoing study evaluating its safety and efficacy in healthy children younger than 1 year of age (ClinicalTrials.gov Identifier: NCT03653364) and a study to assess the potential to reduce transmission of influenza from an infected patient to healthy individuals (ClinicalTrials.gov Identifier: NCT03969212).

In otherwise healthy adults and adolescents (older than 12 years of age) with uncomplicated influenza, single-dose baloxavir was superior to placebo in alleviating symptoms (median, 53.7 hours [95% CI, 49.5 to 58.5 hours] vs 80.2 hours [95% CI, 72.6 to 87.1 hours]; *P*<.001) and superior to oseltamivir and placebo in reducing viral load and time to cessation of viral shedding (significant reductions observed by day 2 of treatment) in the double-blind, controlled, phase 3 CAPSTONE-1 trial.^48^ Adverse events were reported in 20.7%, 24.8%, and 24.6% of patients taking baloxavir, oseltamivir, and placebo, respectively. Adverse events that were considered to be related to the trial regimen were more common in oseltamivir recipients (8.4%) compared with baloxavir (4.4%; *P*=.009) or placebo recipients (3.9%). Two serious adverse events reported in baloxavir recipients (incarcerated inguinal hernia and aseptic meningitis) were considered to be unrelated to baloxavir.

Single-dose baloxavir was superior to placebo (difference in median time to improvement of symptoms of 29.1 hours [95% CI, 14.6 to 42.8 hours]; *P*<.0001) and similar to oseltamivir (difference in median time to improvement of symptoms of 7.7 hours [95% CI, −7.9 to 22.7 hours]) in ameliorating influenza symptoms in high-risk adult and adolescent outpatients diagnosed with influenzalike illness in the double-blind, randomized, controlled, phase 3 CAPSTONE-2 trial.^49^ Of note, as early as day 1 after treatment initiation, baloxavir was associated with a significantly faster decline in infectious virus titer compared with oseltamivir and placebo. In addition, baloxavir was associated with significantly fewer influenza-related complications than placebo and numerically fewer than oseltamivir.^49^ Adverse events were reported in 25% of baloxavir recipients compared with 30% of placebo and 28% of oseltamivir recipients, with the most frequently identified adverse events being bronchitis, sinusitis, diarrhea, and nausea. Adverse events leading to withdrawal of the study drug were pneumonia (baloxavir, n=2; oseltamivir, n=1), vomiting (baloxavir, n=2), and bronchitis (placebo, n=2). Serious adverse events were reported in all treatment groups: baloxavir (n=5), placebo (n=9), and oseltamivir (n=8). Of these adverse events, 1 case of hypertension and 1 case of nausea in the placebo group and 2 cases of elevations in transaminase levels in the oseltamivir group were considered to be treatment related.

In otherwise healthy children with influenza aged 1 to younger than 12 years, single-dose baloxavir had similar safety (primary end point; adverse events reported in 46.1% and 53.4% of patients, respectively) and efficacy to oseltamivir in alleviating symptoms of influenza, with a median time to alleviation of symptoms of 138.1 hours (95% CI, 116.6 to 163.2 hours) and 150.0 hours (95% CI, 115.0 to 165.7 hours), respectively, in the randomized, double-blind, phase 3 miniSTONE-2 trial.^50^ The overall incidence of adverse events was similar between baloxavir (46.1%) and oseltamivir (53.4%). The incidence of treatment-related adverse events was low in both groups, 2.6% vs 8.6% for baloxavir compared with oseltamivir, respectively. The most common adverse events in both groups were gastrointestinal disorders (vomiting or diarrhea), experienced by 10.4% and 17.2% of patients, respectively. No deaths, serious adverse events, or hospitalizations were reported during the study.

### Resistance to Antiviral Agents

All antiviral drugs exert a selective pressure on a virus, which can result in the emergence of new viruses resistant to the antiviral. Such treatment-emergent resistance is, therefore, a natural and expected consequence of antiviral treatment and is known for all antiviral classes.^63^

Influenza viruses change over time, and factors such as virus type or subtype, emergence of resistance, or changes in viral virulence could diminish the clinical benefit of antiviral drugs.^64^ Influenza viruses can have reduced susceptibility to one or more influenza antiviral agents. Reduced susceptibility to antivirals, which may be an indication of potential antiviral treatment-emergent resistance, occurs when an influenza virus changes the antiviral binding at the active site.

Consequently, some antiviral agents may not be as effective in the treatment of viruses with reduced susceptibility.^65^ High levels of resistance continue to be observed with amantadine and rimantadine (>99%) among circulating influenza A/H3N2 and A/H1N1pdm09 viruses. Therefore, these drugs are no longer recommended against currently circulating influenza A viruses.

To date, the majority of data regarding treatment-emergent resistance to neuraminidase inhibitors has been obtained with oseltamivir because it has been available for decades and is the most widely used neuraminidase inhibitor. Resistance to oseltamivir first emerged during the 2007-2008 influenza season.^46^^,^^47^ In the subsequent influenza seasons, oseltamivir resistance rates have varied and are higher for the H1 virus subtype. Resistance rates have been reported in 1% to 6% of adults and adolescents and in 2% to 37% of children.^66^^,^^67^ However, it is important to note that these are rates of treatment-emergent resistance, ie, resistance arising in the virus of an infected individual under drug selection pressure, and do not give an indication of whether these variants will be sufficiently fit to transmit and spread in the population. Indeed, rates of circulating (or “acquired”) resistance tend to be much lower (discussed subsequently).

Influenza viruses with reduced in vitro susceptibility to baloxavir due to virus mutations and amino acid substitutions in viral proteins sometimes emerge. The most common viral treatment–emergent substitutions during baloxavir treatment are in position 38 of polymerase acid protein.^68^ Reduced susceptibility to baloxavir attributed to PA/I38X substituted virus has been reported in clinical trials in up to 9.7% of adolescents and adults^48^^,^^69^ and 23.4% of children.^50^^,^^70^ There has been no indication that treatment-emergent baloxavir-resistant viruses meaningfully alter the normal clinical course of influenza infection, and it is important to reiterate that, similar to the case for oseltamivir, these treatment-emergent rates do not reflect rates of circulating resistance. However, additional studies are needed to understand the clinical impact of these treatment-emergent viral substitutions.

Direct comparisons between oseltamivir and baloxavir treatment-emergent resistance rates should not be made because of the differences in study design, study populations, and methodologies for viral detection. In addition, it is important to distinguish treatment-emergent resistance rates from rates of circulating resistant, which refers to a virus that is already resistant at the point of infection. Antiviral resistance and reduced susceptibility to the neuraminidase inhibitors and to baloxavir among circulating influenza viruses is currently very low (<1% and <0.1%, respectively, according to the most recent World Health Organization reports),^71^, ^72^, ^73^ and the CDC and the World Health Organization will continue to monitor the occurrence of resistance.^74^ In summary, the benefits of antiviral treatment far outweigh concerns of potential resistance, which in the vast majority of cases does not have a significant clinical impact.^63^

## Treatment Recommendations

As of January 25, 2021, the CDC recommends antiviral treatment as soon as possible (≤48 hours) for any patient with confirmed or suspected influenza who is hospitalized, has severe, complicated, or progressive illness, or is at higher risk for influenza complications, including young children, adults 65 years of age and older, and people with comorbidities, eg, asthma, diabetes, and heart disease.^17^ Decisions regarding initiation of antiviral treatment should not be delayed for laboratory confirmation of influenza in priority patients. The CDC has also produced a guide for considering influenza testing and treatment when influenza viruses are circulating in the community regardless of influenza vaccination history (Figure 1 and Supplemental Appendix). This algorithm may be especially valuable for primary care professionals managing influenza during the current COVID-19 pandemic. In addition, the CDC notes that antivirals can be considered as a treatment option for uncomplicated influenza infection in patients who are otherwise healthy, if treatment can be initiated within 48 hours.^17^

For outpatients with suspected or confirmed uncomplicated influenza, oral oseltamivir, inhaled zanamivir, intravenous peramivir, or single oral baloxavir may be used for treatment, depending on approved age groups and contraindications (Table 3).^17^^,^^78^ For hospitalized patients with suspected or confirmed influenza, the most recent CDC recommendations advocate the initiation of antiviral treatment with oral or enterally administered oseltamivir as soon as possible.^17^ For outpatients with complications or progressive disease and suspected or confirmed influenza (eg, pneumonia or exacerbation of underlying chronic medical conditions), initiation of antiviral treatment with oral oseltamivir is recommended as soon as possible.^17^

**Table 3:** Antivirals Recommended by the Centers for Disease Control and Prevention for the Treatment and Prophylaxis of Influenza A and B

Antiviral	Treatment		Prophylaxis		Limitations
	Indication/usage	Dose	Indication/usage	Dose	
Polymerase acidic endonuclease inhibitor					
Baloxavir^75^	• ≥12 y (uncomplicated influenza; symptomatic for ≤2 d) • Otherwise healthy • High risk for influenza complications	Tablets: • Less than 80 kg: one 40-mg tablet • At least 80 kg: one 80- mg tablet Oral suspension: • Less than 80 kg: 40 mg/20 mL (1 bottle) taken as a single dose • At least 80 kg: 80 mg/40 mL (2 bottles) taken as a single dose	• Postexposure prophylaxis of influenza in patients ≥12 y following contact with an individual who has influenza	Tablets: • Less than 80 kg: one 40-mg tablet • At least 80 kg: one 80-mg tablet Oral suspension: • Less than 80 kg: 40 mg/20 mL (1 bottle) taken as a single dose • At least 80 kg: 80 mg/40 mL (2 bottles) taken as a single dose	• Consider available information on drug susceptibility patterns for circulating influenza virus strains when deciding whether to use
Neuraminidase inhibitors					
Oseltamivir^66^	• ≥2 y (uncomplicated influenza; symptomatic for ≤2 d)	• Adults and adolescents (≥13 y): 75 mg twice daily orally for 5 d • Pediatric patients (1-12 y): based on weight twice daily for 5 d • Pediatric patients (2 wk to <1 y): 3 mg/kg twice daily for 5 d	• ≥1 y	• Adults and adolescents (>13 y): 75 mg once daily for ≥10 d • Community outbreak: 75 mg once daily for ≤6 wk • Pediatric patients (1-12 y): based on weight once daily for 10 d • Community outbreak: based on weight once daily for ≤6 wk	• Efficacy not established in patients who begin therapy after 48 h of symptoms
Peramivir^76^	• ≥2 y	• Adults and adolescents (≥13 y): 600 mg IV (≤2 d of symptom onset) • Pediatric patients (2-12 y): 12 mg/kg IV (≤2 d of symptom onset; up to 600 mg)	• Not recommended		• Limited data in influenza B • Efficacy could not be established in serious influenza requiring hospitalization
Zanamivir^77^	• ≥7 y (symptomatic for ≤2 d)	• 10 mg inhaled twice daily for 5 d	• ≥5 y	• Household contact: 10 mg inhaled once daily for 10 d • Community outbreak: 10 mg inhaled once daily for 28 d	• Underlying respiratory disease (eg, asthma, COPD)

COPD, chronic obstructive pulmonary disease; IV, intravenous.

## Antivirals: Impact on Transmission and Prophylaxis

Household contacts is one of the highest-risk groups for exposure given their proximity. Once a household member is infected with influenza, the risk of onward transmission within the household has been estimated to be 38%.^7^^,^^79^ Transmission of influenza within households suggests that children play a significant role in the transmission of influenza within families, with 40% to 48% of secondary cases exposed to a child with influenza attributable to transmission from the child.^29^ Median lag time between influenza onset in the index patient and symptom onset in the secondary patient was 2 days (range, 1 to 5 days).^29^ Household transmission studies also suggest that the rate of transmission from an infectious household to the wider community is 20% and 13% of unvaccinated and vaccinated individuals, respectively.^80^ Prompt treatment of infected index patients with antivirals could potentially be a strategy for reducing influenza transmission to household contacts, as discussed by Hayden et al.^81^ Another strategy is postexposure prophylaxis in individuals who come into contact with a patient with influenza within the household setting, which may be particularly important in individuals at high risk for complications.

Prophylaxis has the potential to prevent an estimated 20% to 40% of secondary cases of household influenza caused by exposure to a household member with influenza.^29^ Furthermore, if less than 48 hours has elapsed since symptom onset in children with influenza, then prophylaxis of contacts can be initiated with an efficacy rate of up to 89% in preventing further spread of the disease.

Antivirals can be used for prophylaxis, but they should not be considered a substitute for primary prevention of influenza infection via vaccination. However, they can be used prophylactically, either preexposure or postexposure, per the approved label. The prophylactic use of antivirals can particularly benefit individuals at high risk for complications who have been exposed to influenza within 2 weeks of vaccination, high-risk individuals who are immunocompromised or cannot be vaccinated because of contraindications, or as part of a strategy to control institutional outbreaks.^17^

The neuraminidase inhibitors oseltamivir and zanamivir have been reported to be effective for preexposure and postexposure prophylaxis, providing up to 89% (95% CI, 67% to 97%; *P*<.001) protection from influenza infection among individuals.^82^ Zanamivir is indicated for the prophylaxis of influenza in patients 5 years of age and older^77^ and oseltamivir in patients 1 year of age and older.^66^

Baloxavir has also shown efficacy as postexposure prophylaxis for influenza in household contacts of patients with influenza. In a double-blind, randomized, controlled trial, postexposure single-dose baloxavir treatment was significantly more effective than placebo in preventing influenza infection among household contacts of patients with influenza (influenza developed in 1.9% vs 13.6% of contacts, respectively; adjusted risk ratio, 0.14 [95% CI, 0.06 to 0.30]; *P*<.001).^51^

The benefit of early intervention is not trivial. A recent study that modeled the impact of transmission from clinical trial data on reduction of viral load and duration of illness of infected individuals concluded that approximately 22 million infections and more than 6000 deaths would have been averted in the 2017-2018 influenza season by administering baloxavir to 30% of infected individuals within 48 hours after symptom onset.^83^ Moreover, if treatment were initiated within 24 hours, the impact would have increased by approximately 2-fold.^84^

## Summary and Conclusions

Currently, the CDC is maximizing influenza vaccination efforts during the 2021-2022 influenza season, given the likely coexistence of influenza and COVID-19. This effort includes purchasing an additional 2 million doses of pediatric and 9.3 million doses of adult influenza vaccine, emphasizing the importance of preparing for the influenza season, and targeting community outreach to groups at high risk for influenza. These groups are often at high risk for COVID-19.^31^ The known variability in the effectiveness of influenza vaccination at both the individual and population level underscores the need for effective antivirals for the management of influenza. In addition, as of January 25, 2021, the CDC has updated its recommendations on antiviral treatment and recommends empirical antiviral treatment as early as possible for any patient who is hospitalized, has severe, complicated, or progressive illness;, or is at higher risk for influenza complications during periods of community cocirculation of influenza and SARS-CoV-2.^17^

Treatment with antivirals will be imperative in decreasing the burden of influenza, especially in the context of COVID-19.^12^^,^^26^^,^^85^^,^^86^ In outpatients, choice of antiviral treatment should take into consideration patient age, health status, and risk factors or comorbidities, along with patient adherence. The role of patient adherence in successful antiviral treatment for influenza should not be underestimated; route of administration, specifically oral in preference to inhalation, appears to drive patient adherence to a certain degree.^87^ For this reason, oral treatment may be preferable to inhalation during the influenza season. A single-dose treatment option makes “directly observed therapy” possible for the entire treatment course. Baloxavir has similar efficacy against both influenza A and influenza B infections, provides full effect after a single oral dose, and is effective against key neuraminidase-resistant influenza strains.^46^

Households represent a critical setting for transmission of influenza, given the close proximity to infected patients shedding infectious virus.^79^ Prophylactic use of antivirals has a positive impact on the prevention of influenza infection in healthy household contacts of influenza-infected patients. Both oseltamivir and zanamivir are FDA-approved for prophylaxis against influenza within the household setting and for community outbreaks. Baloxavir is effective for postexposure prophylaxis, and baloxavir treatment has been found to be more effective than placebo for postexposure prophylaxis of influenza in patients following contact with a person who has influenza.^51^ In late November 2020, baloxavir marboxil was approved by the FDA for postexposure prophylaxis in patients 12 years of age and older.^75^ Preclinical data have shown that baloxavir reduced infectious viral shedding in the upper respiratory tract compared with placebo and reduced the frequency of transmission among sentinels, even when treatment was delayed until 2 days following infection.^88^ Additional clinical trials are currently being conducted to assess the efficacy of baloxavir in reducing onward transmission of influenza A or B in households (ClinicalTrials.gov Identifier: NCT03969212).

## References

[1] Gaitonde D.Y., Moore F.C., Morgan M.K. (2019). Influenza: diagnosis and treatment. Am Fam Physician.

[2] Centers for Disease Control and Prevention Flu symptoms & complications. https://www.cdc.gov/flu/symptoms/symptoms.htm.

[3] Leung N.H.L., Xu C., Ip D.K.M., Cowling B.J. (2015). The fraction of influenza virus infections that are asymptomatic: a systematic review and meta-analysis. Epidemiology.

[4] Boktor S.W., Hafner J.W. (2021). *StatPearls* [internet].

[5] Centers for Disease Control and Prevention About flu. https://www.cdc.gov/flu/about/index.html.

[6] Grech V., Borg M. (2020). Influenza vaccination in the COVID-19 era. Early Hum Dev.

[7] Centers for Disease Control and Prevention People at high risk for flu complications. https://www.cdc.gov/flu/highrisk/index.htm.

[8] Erlikh I.V., Abraham S., Kondamudi V.K. (2010). Management of influenza. Am Fam Physician.

[9] Centers for Disease Control and Prevention Disease burden of influenza. https://www.cdc.gov/flu/about/burden/index.html.

[10] Thompson W.W., Shay D.K., Weintraub E. (2003). Mortality associated with influenza and respiratory syncytial virus in the United States. JAMA.

[11] Zhou H., Thompson W.W., Viboud C.G. (2012). Hospitalizations associated with influenza and respiratory syncytial virus in the United States, 1993-2008. Clin Infect Dis.

[12] Putri W.C.W.S., Muscatello D.J., Stockwell M.S., Newall A.T. (2018). Economic burden of seasonal influenza in the United States. Vaccine.

[13] Grohskopf L.A., Alyanak E., Broder K.R. (2020). Prevention and control of seasonal influenza with vaccines: recommendations of the Advisory Committee on Immunization Practices - United States, 2020-21 influenza season. MMWR Recomm Rep.

[14] Dini G., Toletone A., Sticchi L., Orsi A., Bragazzi N.L., Durando P. (2018). Influenza vaccination in healthcare workers: a comprehensive critical appraisal of the literature. Hum Vaccin Immunother.

[15] Centers for Disease Control and Prevention Similarities and differences between flu and COVID-19. https://www.cdc.gov/flu/symptoms/flu-vs-covid19.htm.

[16] Zayet S., Kadiane-Oussou N.J., Lepiller Q. (2020). Clinical features of COVID-19 and influenza: a comparative study on Nord Franche-Comte cluster. Microbes Infect.

[17] Centers for Disease Control and Prevention Influenza antiviral medications: summary for clinicians. https://www.cdc.gov/flu/professionals/antivirals/summary-clinicians.htm#Treatment.

[18] Centers for Disease Control and Prevention 2019-2020 U.S. flu season: preliminary in-season burden estimates. https://www.cdc.gov/flu/about/burden/preliminary-in-season-estimates.htm.

[19] Reed C., Chaves S.S., Daily Kirley P. (2015). Estimating influenza disease burden from population-based surveillance data in the United States. PLoS One.

[20] Olsen S.J., Azziz-Baumgartner E., Budd A.P. (2020). Decreased influenza activity during the COVID-19 pandemic — United States, Australia, Chile, and South Africa, 2020. MMWR Morb Mortal Wkly Rep.

[21] Leung N.H.L., Chu D.K.W., Shiu E.Y.C. (2020). Respiratory virus shedding in exhaled breath and efficacy of face masks. Nat Med.

[22] Centers for Disease Control and Prevention Interim guidance for the use of masks to control seasonal influenza virus transmission: guidelines and recommendations. https://www.cdc.gov/flu/professionals/infectioncontrol/maskguidance.htm.

[23] World Health Organization How to wear a medical mask safely. https://www.who.int/emergencies/diseases/novel-coronavirus-2019/advice-for-public/when-and-how-to-use-masks.

[24] World Health Organization How to wear a non-medical fabric mask safely. https://www.who.int/images/default-source/health-topics/coronavirus/clothing-masks-infographic---(web)-logo-who.png?sfvrsn=b15e3742_16.

[25] Immunization Action Coalition (2020). https://www.immunize.org/askexperts/experts_inf.asp.

[26] Rolfes M.A., Foppa I.M., Garg S. (2018). Annual estimates of the burden of seasonal influenza in the United States: a tool for strengthening influenza surveillance and preparedness. Influenza Other Respir Viruses.

[27] Centers for Disease Control and Prevention Selecting viruses for the seasonal influenza vaccine. https://www.cdc.gov/flu/prevent/vaccine-selection.htm.

[28] Centers for Disease Control and Prevention Flu vaccination coverage, United States, 2019–20 influenza season. https://www.cdc.gov/flu/fluvaxview/coverage-1920estimates.htm.

[29] Viboud C., Boëlle P.-Y., Cauchemez S. (2004). Risk factors of influenza transmission in households. Br J Gen Pract.

[30] Baguelin M., Flasche S., Camacho A., Demiris N., Miller E., Edmunds W.J. (2013). Assessing optimal target populations for influenza vaccination programmes: an evidence synthesis and modelling study. PLoS Med.

[31] Centers for Disease Control and Prevention 2020-2021 Flu season summary FAQ. https://www.cdc.gov/flu/season/faq-flu-season-2020-2021.htm.

[32] Centers for Disease Control and Prevention Influenza prevention: information for travelers. https://www.cdc.gov/flu/school-business/travelersfacts.htm.

[33] Merckx J., Wali R., Schiller I. (2017). Diagnostic accuracy of novel and traditional rapid tests for influenza infection compared with reverse transcriptase polymerase chain reaction: a systematic review and meta-analysis. Ann Intern Med.

[34] Centers for Disease Control and Prevention Rapid influenza diagnostic tests. https://www.cdc.gov/flu/professionals/diagnosis/clinician_guidance_ridt.htm.

[35] Centers for Disease Control and Prevention Influenza virus testing methods. https://www.cdc.gov/flu/professionals/diagnosis/table-testing-methods.htm.

[36] Mallefet P., Maret S., Fry S. (2020). A prospective, multicenter study to evaluate the performance of a novel home diagnostic kit for detection of influenza A and B [abstract]. Am J Respir Crit Care Med.

[37] Primary Care Innovation Lab. Flu@home – self testing for influenza. https://familymedicine.uw.edu/pci-lab/projects/hometest.testing/fluhome-self-testing-for-influenza.

[38] Green D.A., StGeorge K. (2018). Rapid antigen tests for influenza: rationale and significance of the FDA reclassification. J Clin Microbiol.

[39] Centers for Disease Control and Prevention Overview of influenza testing methods. https://www.cdc.gov/flu/professionals/diagnosis/overview-testing-methods.htm.

[40] Centers for Disease Control and Prevention Table 3. Nucleic Acid Detection Based Tests. https://www.cdc.gov/flu/professionals/diagnosis/table-nucleic-acid-detection.html.

[41] Hoady R. (September 3, 2020).

[42] Nakajima M. (September 14, 2020).

[43] Centers for Disease Control and Prevention Table 4. Multiplex Assays Authorized for Simultaneous Detection of Influenza Viruses and SARS-CoV-2 by FDA. https://www.cdc.gov/flu/professionals/diagnosis/table-flu-covid19-detection.html.

[44] Krejcova L., Michalek P., Hynek D., Adam V., Kizek R. (2015). Structure of influenza viruses, connected with influenza life cycle. J Metallomics Nanotechnol.

[45] McKimm-Breschkin J.L. (2013). Influenza neuraminidase inhibitors: antiviral action and mechanisms of resistance. Influenza Other Respir Viruses.

[46] Toots M., Plemper R.K. (2020). Next-generation direct-acting influenza therapeutics. Transl Res.

[47] Krammer F., Smith G.J.D., Fouchier R.A.M. (2018). Influenza. Nat Rev Dis Primers.

[48] Hayden F.G., Sugaya N., Hirotsu N., Baloxavir Marboxil Investigators Group (2018). Baloxavir marboxil for uncomplicated influenza in adults and adolescents. N Engl J Med.

[49] Ison M.G., Portsmouth S., Yoshida Y. (2020). Early treatment with baloxavir marboxil in high-risk adolescent and adult outpatients with uncomplicated influenza (CAPSTONE-2): a randomised, placebo-controlled, phase 3 trial. Lancet Infect Dis.

[50] Baker J., Block S.L., Matharu B. (2020). Baloxavir marboxil single-dose treatment in influenza-infected children: a randomized, double-blind, active controlled phase 3 safety and efficacy trial (miniSTONE-2). Pediatr Infect Dis J.

[51] Ikematsu H., Hayden F.G., Kawaguchi K. (2020). Baloxavir marboxil for prophylaxis against influenza in household contacts. N Engl J Med.

[52] Treanor J.J., Hayden F.G., Vrooman P.S., US Oral Neuraminidase Study Group (2000). Efficacy and safety of the oral neuraminidase inhibitor oseltamivir in treating acute influenza: a randomized controlled trial. JAMA.

[53] Hayden F.G., Treanor J.J., Fritz R.S. (1999). Use of the oral neuraminidase inhibitor oseltamivir in experimental human influenza: randomized controlled trials for prevention and treatment. JAMA.

[54] Kohno S., Kida H., Mizuguchi M., Shimada J., S-021812 Clinical Study Group (2010). Efficacy and safety of intravenous peramivir for treatment of seasonal influenza virus infection. Antimicrob Agents Chemother.

[55] de Jong M.D., Ison M.G., Monto A.S. (2014). Evaluation of intravenous peramivir for treatment of influenza in hospitalized patients. Clin Infect Dis.

[56] Monto A.S., Webster A., Keene O. (1999). Randomized, placebo-controlled studies of inhaled zanamivir in the treatment of influenza A and B: pooled efficacy analysis. J Antimicrob Chemother.

[57] Hedrick J.A., Barzilai A., Behre U. (2000). Zanamivir for treatment of symptomatic influenza A and B infection in children five to twelve years of age: a randomized controlled trial. Pediatr Infect Dis J.

[58] LaForce C., Man C.Y., Henderson F.W. (2007). Efficacy and safety of inhaled zanamivir in the prevention of influenza in community-dwelling, high-risk adult and adolescent subjects: a 28-day, multicenter, randomized, double-blind, placebo-controlled trial. Clin Ther.

[59] Muthuri S.G., Venkatesan S., Myles P.R., PRIDE Consortium Investigators (2014). Effectiveness of neuraminidase inhibitors in reducing mortality in patients admitted to hospital with influenza A H1N1pdm09 virus infection: a meta-analysis of individual participant data. Lancet Respir Med.

[60] Dobson J., Whitley R.J., Pocock S., Monto A.S. (2015). Oseltamivir treatment for influenza in adults: a meta-analysis of randomised controlled trials [published corrections appear in *Lancet*. 2015;385(9979):1728]. Lancet.

[61] Venkatesan S., Myles P.R., Leonardi-Bee J. (2017). Impact of outpatient neuraminidase inhibitor treatment in patients infected with influenza A(H1N1)pdm09 at high risk of hospitalization: an individual participant data metaanalysis. Clin Infect Dis.

[62] Kumar D., Ison M.G., Mira J.P. (2021). Combining baloxavir with standard-of-care neuraminidase inhibitor in patients hospitalised with severe influenza (FLAGSTONE): a randomised, parallel-group, double-blind, placebo-controlled, superiority trial. Lancet Infect Dis.

[63] Holmes E.C., Hurt A.C., Dobbie Z., Clinch B., Oxford J.S., Piedra P.A. (2021). Understanding the impact of resistance to influenza antivirals. Clin Microbiol Rev.

[64] (2018). Xofluza [package insert].

[65] Centers for Disease Control and Prevention Influenza antiviral drug resistance. https://www.cdc.gov/flu/treatment/antiviralresistance.htm.

[66] (2012). Tamiflu [package insert].

[67] Lina B., Boucher C., Osterhaus A. (2018). Five years of monitoring for the emergence of oseltamivir resistance in patients with influenza A infections in the Influenza Resistance Information Study. Influenza Other Respir Viruses.

[68] Gubareva L.V., Fry A.M. (2020). Baloxavir and treatment-emergent resistance: public health insights and next steps [editorial]. J Infect Dis.

[69] Uehara T., Hayden F.G., Kawaguchi K. (2020). Treatment-emergent influenza variant viruses with reduced baloxavir susceptibility: impact on clinical and virologic outcomes in uncomplicated influenza. J Infect Dis.

[70] Hirotsu N., Sakaguchi H., Sato C. (2020). Baloxavir marboxil in Japanese pediatric patients with influenza: safety and clinical and virologic outcomes. Clin Infect Dis.

[71] World Health Organization Recommended composition of influenza virus vaccines for use in the 2021 Southern Hemisphere influenza season. https://www.who.int/influenza/vaccines/virus/recommendations/202009_recommendation.pdf?ua=1.

[72] World Health Organization Recommended composition of influenza virus vaccines for use in the 2020-2021 Northern Hemisphere influenza season. https://www.who.int/influenza/vaccines/virus/recommendations/202002_recommendation.pdf?ua=1.

[73] World Health Organization Recommended composition of influenza virus vaccines for use in the 2021-2022 Northern Hemisphere influenza season. https://www.who.int/influenza/vaccines/virus/recommendations/202102_recommendation.pdf.

[74] Centers for Disease Control and Prevention Weekly U.S. influenza surveillance report. https://www.cdc.gov/flu/weekly/index.htm.

[75] (2020). Xofluza [package insert].

[76] (September 2017). Rapivab [package insert].

[77] (June 2018). Relenza [package insert].

[78] Centers for Disease Control and Prevention Flu treatment. https://www.cdc.gov/flu/treatment/index.html.

[79] Tsang T.K., Lau L.L.H., Cauchemez S., Cowling B.J. (2016). Household transmission of influenza virus. Trends Microbiol.

[80] Ainslie K.E.C., Haber M.J., Malosh R.E., Petrie J.G., Monto A.S. (2018). Maximum likelihood estimation of influenza vaccine effectiveness against transmission from the household and from the community. Stat Med.

[81] Hayden F.G., Asher J., Cowling B.J. Reducing influenza virus transmission: the value of antiviral treatment. 10.1093/cid/ciab625.

[82] Okoli G.N., Otete H.E., Beck C.R., Nguyen-Van-Tam J.S. (2014). Use of neuraminidase inhibitors for rapid containment of influenza: a systematic review and meta-analysis of individual and household transmission studies. PloS One.

[83] Du Z., Nugent C., Galvani A.P., Krug R.M., Meyers L.A. (2020). Modeling mitigation of influenza epidemics by baloxavir. Nat Commun.

[84] Komeda T., Takazono T., Hosogaya N. (2021). Comparison of household transmission of influenza virus from index patients treated with baloxavir marboxil or neuraminidase inhibitors: a health insurance claims database study. Clin Infect Dis.

[85] Molinari N.-A.M., Ortega-Sanchez I.R., Messonnier M.L. (2007). The annual impact of seasonal influenza in the US: measuring disease burden and costs. Vaccine.

[86] Buchy P., Badur S. (2020). Who and when to vaccinate against influenza. Int J Infect Dis.

[87] Flicoteaux R., Protopopescu C., Tibi A. (2017). Factors associated with non-persistence to oral and inhaled antiviral therapies for seasonal influenza: a secondary analysis of a double-blind, multicentre, randomised clinical trial. BMJ Open.

[88] Lee L.Y.Y., Zhou J., Frise R. (2020). Baloxavir treatment of ferrets infected with influenza A(H1N1)pdm09 virus reduces onward transmission. PLoS Pathog.

