# Supplementary material for: Influenza Management During the COVID-19 Pandemic: A Review of Recent Innovations in Antiviral Therapy and Relevance to Primary Care Practice
Source: Mayo Clin Proc Innov Qual Outcomes. 2021 Aug 14;5(6):974–91. doi: 10.1016/j.mayocpiqo.2021.07.005 (PMC8363430; doi:10.1016/j.mayocpiqo.2021.07.005)
Supplement: Supplemental Table [file mmc1.pdf]

Supplementary Appendix. Footnotes to accompany the CDC treatment algorithm in Figure 1. These are freely available at https://www.cdc.gov/flu/professionals/diagnosis/consider-influenza-testing.htm and their use here does not imply endorsement of this review article by the Centers for Disease Control and Prevention.

**Figure 1. Footnotes to the algorithm for influenza testing during an influenza community outbreak based on the Centers for Disease Control and Prevention Algorithm[1]**
1. Confirmation of influenza virus infection by diagnostic testing is not required for decisions to prescribe antiviral medication. Decision-making should be based upon signs and symptoms consistent with influenza illness and epidemiological factors. Initiation of empirical antiviral treatment should not be delayed while influenza testing results are pending. Antiviral treatment is clinically most beneficial when started as close as possible to illness onset. Influenza vaccine effectiveness is moderate, and therefore, a history of current-season influenza vaccination does not exclude a diagnosis of influenza.
2. Signs and symptoms of uncomplicated influenza vary by age, underlying health conditions, and immune function. Common signs and symptoms include fever with nonproductive cough or other suggestive respiratory symptoms, often with myalgias or headache. Fever is not always present, including in premature and young infants, immunocompromised and immunosuppressed persons, and especially in elderly persons. Note that some persons may have atypical presentations—especially infants (eg, sepsis-like syndrome) and the elderly (eg, confusion).
3. Complications associated with influenza can vary by age, immune status, and underlying medical conditions. Some examples include worsening of underlying chronic medical

conditions (eg, worsening of congestive cardiac failure; asthma exacerbation; exacerbation of chronic obstructive pulmonary disease); lower respiratory tract disease (pneumonia, bronchiolitis, croup, respiratory failure); invasive bacterial co-infection; cardiac (eg, myocarditis); musculoskeletal (eg, myositis, rhabdomyolysis); neurologic (eg, encephalopathy, encephalitis); multi-organ failure (septic shock, renal failure, respiratory failure).
4. Influenza testing may be used to inform decisions on the use of antiviral treatment, the use of antibiotic treatment, the need for further diagnostic tests, consideration for home care, or recommendations for ill persons living with others who are at high risk for influenza complications. Proper interpretation of influenza testing results must consider a number of factors, including the following: the predictive values of the test, test sensitivity and specificity compared with a "gold standard" test, prevalence of influenza in the patient population, time from illness onset to specimen collection and whether the person may still have detectable influenza viral shedding, and source of the respiratory specimen (upper or lower respiratory tract). To maximize the detection of influenza viruses, respiratory specimens should be collected as close as possible to illness onset (ideally <3-4 days after onset; molecular assays may detect influenza viral RNA in respiratory tract specimens for longer periods after illness onset than antigen detection assays). See this algorithm for more information. The Infectious Diseases Society of America (IDSA) recommends the use of rapid influenza molecular assays over rapid influenza diagnostic tests (RIDTs) for the detection of influenza viruses in respiratory specimens of outpatients. Consult the IDSA Influenza Clinical Practice Guidelines external icon for recommendations on influenza testing and interpretation of testing results. Consult guidance on antibiotic use from the IDSA, American Thoracic Society, and the American Academy of Pediatrics. Antiviral treatment is recommended as soon as possible for

hospitalized patients with suspected influenza without waiting for influenza testing results of molecular assays. Guidance on antiviral treatment of influenza is available.
5. All hospitalized patients with suspected influenza should be tested with molecular assays with high sensitivity and specificity (eg, reverse transcription polymerase chain reaction [RT-PCR]) because the detection of influenza virus infection and prompt initiation of antiviral therapy are most clinically beneficial, and prompt implementation of infection prevention and control measures is essential for the prevention of nosocomial influenza outbreaks. The IDSA recommends the use of RT-PCR or other molecular assays for the detection of influenza viruses in respiratory specimens of hospitalized patients. Consult the IDSA Influenza Clinical Practice Guidelines external icon for recommendations on influenza testing and interpretation of testing results. Molecular assays can detect influenza viral nucleic acids in respiratory specimens for longer periods and with much higher accuracy than antigen detection assays. For hospitalized patients with lower respiratory tract disease and suspected influenza, lower respiratory tract specimens should be collected and tested for influenza viruses by RT-PCR because influenza viral shedding in the lower respiratory tract may be detectable for longer periods than in the upper respiratory tract, if influenza testing of upper respiratory tract specimens yields a negative result. If the patient is critically ill on invasive mechanical ventilation, and has tested negative for influenza viruses on an upper respiratory tract specimen, including by a molecular assay, a lower respiratory tract specimen (endotracheal aspirate or bronchioalveolar lavage fluid) should be collected for influenza testing by RT-PCR or other molecular assays. See Prevention Strategies for Seasonal Influenza in Health Care Settings for more information.

6. Influenza testing may help inform decisions on infection prevention and control practices. See Prevention Strategies for Seasonal Influenza in Health Care Settings for more information.
7. Persons who are at Higher Risk of Complications from Influenza include the following: those aged 65 years and older or younger than 2 years of age; pregnant women; those with chronic lung disease (including asthma), heart, renal, metabolic, hematological, and neurological disease; those with immunosuppression; those with morbid obesity; American Indians or Alaska Natives; and residents of chronic care facilities.
8. Antiviral treatment is recommended as soon as possible for outpatients with suspected or confirmed influenza who are at high risk for complications from influenza, or those with progressive disease not requiring hospital admission. Outpatients who are not at higher risk of complications from influenza can be considered based on clinical judgment if presenting within 2 days of illness onset. Guidance on antiviral treatment of influenza is available.

Reference

1. Centers for Disease Control and Prevention. Influenza Antiviral Medications: Summary for Clinicians. Vol 2021. Atlanta, GA: Centers for Disease Control and Prevention; 2021.
